# Pigment Epithelium-Derived Factor (PEDF) is a Determinant of Stem Cell Fate: Lessons from an Ultra-Rare Disease

**DOI:** 10.3390/jdb3040112

**Published:** 2015-11-20

**Authors:** Usman Sagheer, Jingjing Gong, Chuhan Chung

**Affiliations:** 1 Department of Medicine, Yale University School of Medicine, New Haven, CT 06510, USA; 2 VA CT Healthcare System, West Haven, CT 06516, USA

**Keywords:** pigment epithelium-derived factor, osteogenesis imperfecta, stem cells, Wnt signaling

## Abstract

PEDF is a secreted glycoprotein that is widely expressed by multiple organs. Numerous functional contributions have been attributed to PEDF with antiangiogenic, antitumor, anti-inflammatory, and neurotrophic properties among the most prominent. The discovery that null mutations in the PEDF gene results in Osteogenesis Imperfecta Type VI, a rare autosomal recessive bone disease characterized by multiple fractures, highlights a critical developmental function for this protein. This ultra-rare orphan disease has provided biological insights into previous studies that noted PEDF’s effects on various stem cell populations. In addition to bone development, PEDF modulates resident stem cell populations in the brain, muscle, and eye. Functional effects on human embryonic stem cells have also been demonstrated. An overview of recent advances in our understanding by which PEDF regulates stem cells and their potential clinical applications will be evaluated in this review.

## 1. Introduction

Pigment epithelium derived factor (PEDF) is a 50kDa secreted glycoprotein consisting of 418 amino acids that was first identified and isolated from the conditioned medium of cultured human fetal retinal pigment epithelium cells [1,2]. A member of the serine protease inhibitory (SERPIN) superfamily, PEDF lacks protease inhibitory function owing to differences in the reactive center loop (RCL) that characterize other serpins [2,3]. Its gene, *Serpinf1*, is located on chromosome 17p13 and is widely expressed throughout many tissue sites [2,4]. The highest expression levels in the adult human are found in the liver and then adipose tissue [5]. Expression in organs such as the eye, heart, pancreas and others indicate a broad distribution for this protein [4]. PEDF gene expression was reported to be robust in the adult bone [6], but the recent Human Tissue Protein Atlas noted low levels of the PEDF expression in bone [5]. Circulating levels in human sera range from 100 nM in normal weight individuals [7], can reach approximately 500 nM in those with obesity [8], and are undetectable in those with Osteogenesis Imperfecta type VI [9]. In the current paper, we assess the PEDF null state within the context of stem cell biology and examine PEDF’s effects on stem cells in multiple organ sites.

## 2. Osteogenesis Imperfecta Type VI

Osteogenesis imperfecta (OI) type VI is an autosomal recessive disease with marked defects in bone mineralization and multiple fractures [10,11]. OI VI patients do not have fractures at birth, but present clinically starting at six months of age with frequent fractures progressing to bone deformity, vertebral compressions, and scoliosis. Growth deficiency is moderately severe, sclerae are white, and teeth are normal [10]. Bone histology is remarkable for broad bands of un-mineralized osteoid and a fish-scale pattern under polarized light [10]. There is a significantly prolonged mineralization delay seen on quantitative histomorphometry [10,11]. Although overall mineralization is diminished, newer imaging techniques demonstrate focal areas of increased mineralization surrounded by abnormally low mineral content and disorganized collagen fibril assembly, most likely from improper osteoblast-to-osteocyte maturation that affects the early steps of mineralization and assembly of collagen [12]. The murine model of PEDF deficiency closely resembles the bone phenotype seen in humans with excessive accumulation of un-mineralized bone matrix and diminished bone volumes [13].

The causative single gene defect in OI type VI is *Serpinf1*, and encodes for PEDF [9,14–16]. Several truncation mutations in *Serpinf1* have been reported, which result in complete absence of circulating PEDF [9,14]. In addition, homozygous in-frame deletions or insertion mutations in the *Serpinf1* gene cause retention or degradation of PEDF within cellular compartments and results in significantly diminished PEDF secretion with clinical manifestations resembling the null PEDF state [17]. In these circumstances, PEDF levels are >10-fold lower than normal individuals, and their clinical presentation may appear at a more advanced age than in those patients with complete absence of PEDF. From a biological perspective, the single gene deletion of PEDF has provided new insights into the mechanisms of PEDF biology. As Wnt signaling is a major driver of stem cell to osteoblast maturation, a pivotal role for PEDF in modulating Wnt signaling to favor osteoblast differentiation has been demonstrated [18–21]. Studies done outside of bone biology suggest a broader role for PEDF in regulating Wnt signaling [22–25]. This suggests that PEDF’s previously reported diverse biologic effects may stem partly or largely from regulation of this conserved signaling pathway.

## 3. Historical Perspectives & PEDF Signaling

Tombran-Tink and colleagues first identified PEDF in 1991 as a neuronal differentiating factor that could induce retinoblastoma (Rb) cells to adopt morphological and neuronal markers of a more terminally differentiated state [2,26,27]. Treatment of Rb cells with PEDF and *in vivo* transplantation also led to massive tumor growth suggesting that PEDF has context specific effects [28]. This is reflected in the ability of PEDF to promote stem cell renewal, which contrasts sharply with its ability to cause cellular differentiation [29]. Many functional studies, however, indicate a protective role for PEDF including amelioration of glutamate and reactive oxygen species induced toxicity [30–33]. Structurally, disrupting the Serpin loop motif did not remove its neurotrophic function, indicating that PEDF is a non-inhibitory Serpin and that other regions of the protein mediate neuronal differentiation properties [3].

**Figure 1:**
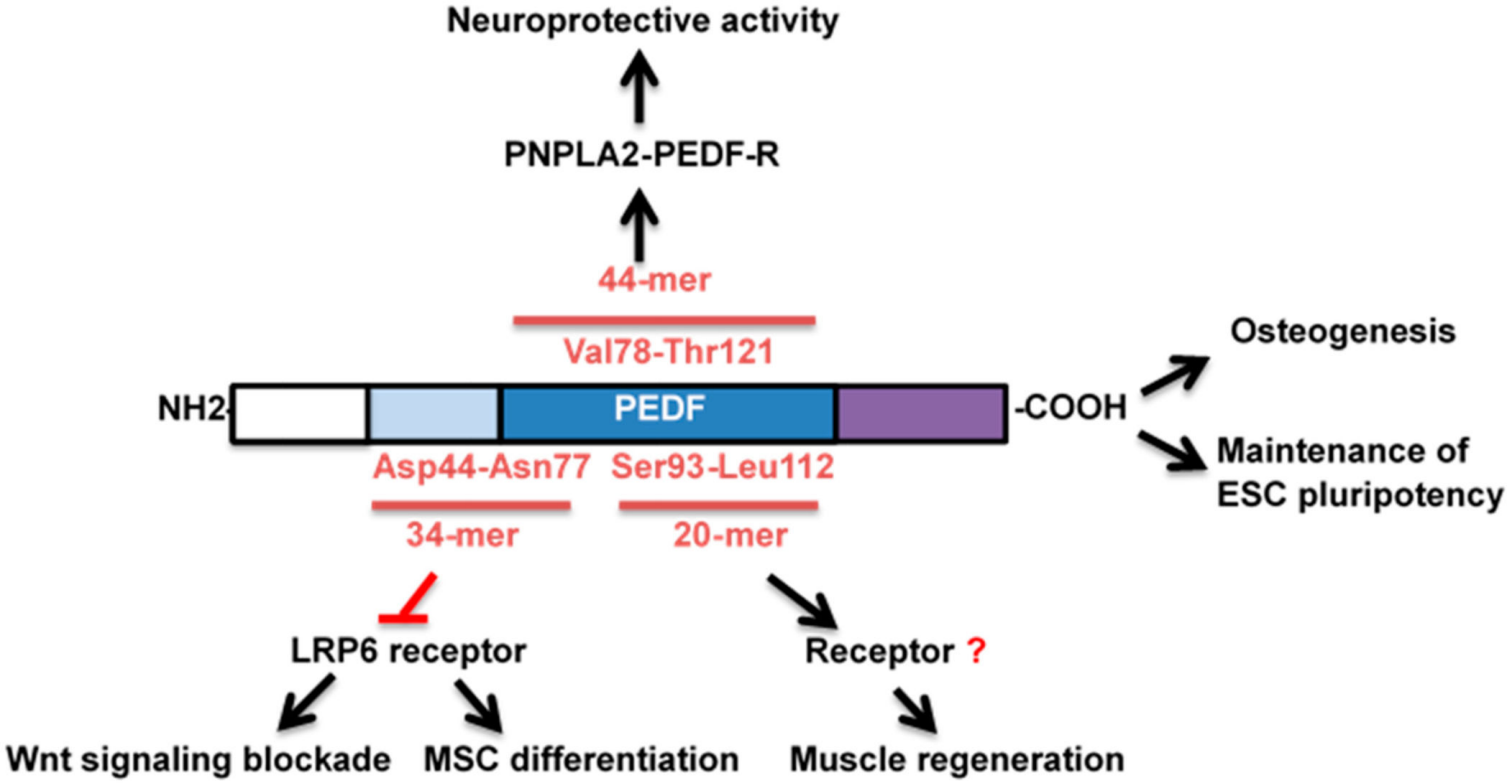
Schematic diagram of PEDF, its associated peptide regions, and functional effects related to stem cell biology. The *N*-terminus contains the well-known anti-angiogenesis effects. This region has also been implicated in inhibiting the Wnt receptor, LRP6, in differentiated cells. The 44-mer and a 20-mer within this region have been implicated in neuronal differentiation and muscle progenitor proliferation. The full-length PEDF protein has been shown to induce mesenchymal stem cells (MSC) to the osteoblast lineage and affect pluripotency of embryonic stem cells (ESC) and apoptosis of inducible pluripotent stem cells.

Based on these earlier discoveries regarding its function as a non-inhibitory Serpin, structure-function relationships were further characterized (Figure 1). Two distinct functional epitopes of PEDF have been identified. A 44-amino-acid fragment of PEDF, corresponding to positions Val78-Thr121 (44-mer) determines neurotrophic activity and binds receptors on the surfaces of different types of neurons [30]. A 34-amino acid fragment mapped to positions Asp44-Asn77 is responsible for PEDF antiangiogenic activity and does not exhibit the neurotrophic activities of the 44-mer PEDF [34]. The regions responsible for stem cell activities have not definitively been identified. One group noted that PEDF 44-mer and a shorter peptide fragment within the 44-mer (Ser93-Leu112) induced regeneration and progenitor cell proliferation in the limbus of rabbits and muscle of rats in response to injury [35,36]. Interestingly, two studies identified Wnt receptor modulation by the 34-mer [23,37] and therefore, the localization of Wnt *vs.* progenitor cell functional motifs at the current time are not considered the same region. Given the role of Wnt signaling in stem cell biology, future studies will need to be conducted to evaluate the effects on stem cell biology by different peptide fragments of PEDF.

Another interpretation of the diverse biological activities of PEDF stems from the multitude of cellular receptors proposed for PEDF and their cell type-specific localization. PNPLA2-PEDF-R, patatin-like phospholipase domain-containing 2 (PNPLA2), binds to 44-mer PEDF and promotes neuronal survival and differentiation by stimulating neuronal surface lipid metabolism [38,39]. Whether this protein, typically associated with the intracellular lipid droplet, is found on the cell surface, however, remains unclear. A laminin receptor, which interacts with the 34-mer, has been shown to be necessary for PEDF-induced inhibition of angiogenesis [40]. A ~60-kDa PEDF-binding protein, cell surface F_1_F_0_-ATP synthase, has been proposed to exert anti-angiogenic and anti-tumorigenic activity by inhibiting cell surface ATP synthase activity [41]. Additional complexity can be examined within the context of PEDF’s ability to bind components of the extracellular matrix including collagen and glycosaminoglycan binding motifs on opposite sides of the protein [42]. Binding to the putative receptors described above, however, does not offer a unifying explanation for the broad functional effects attributed to PEDF.

Ma and colleagues first reported that PEDF is an endogenous antagonist of low density lipoprotein receptor-related protein 6 (LRP6), and blocks Wnt ligand-mediated effects in retinal cells [22]. LRP6 is the membrane-bound co-receptor for the canonical Wnt-β-Catenin pathway (herein “Wnt signaling”). Wnt signaling is a highly conserved pathway that governs nearly every aspect of stem cell maintenance, proliferation and differentiation [43]. After this study, other experimental work confirmed this inhibitory effect on Wnt signaling [23–25,37]. Overexpression of PEDF in a wound-healing model demonstrated that PEDF blocks skin regeneration after induction of wounds through the Wnt signaling blockade [24]. This study used the Wnt reporter mice to demonstrate that systemic PEDF expression nullifies active Wnt signaling at the wound repair site. Although elegant in terms of utilizing a Wnt reporter mouse to demonstrate Wnt signaling blockade *in vivo* by PEDF, these effects do not indicate that PEDF is unnecessary for regenerative processes such as wound repair. Rather, PEDF has specific temporal effects on the different stages of wound repair that are necessary for resolution of the wound-healing process [44]. This is borne out by the finding that PEDF expression and protein levels are low during the early and middle parts of the wound healing process, but undergoes gradual accumulation in the matrix to eventually allow for wound maturation [44]. At the terminal stages of wound repair, PEDF is necessary to terminate Wnt signaling for normal wound repair.

Work from our lab and others corroborate multiple functional outcomes that are consistent with a predominately Wnt inhibitory effect for PEDF. Among these is the inhibition of cellular proliferation and fibrogenic responses in the pancreas and liver that are consistent with previously reported effects of Wnt signaling on tissue fibrosis [23,37,45–47]. Genomic and metabolic profiling of PEDF KO mice identifies other features associated with activated Wnt signaling pathways in the liver [23,48]. Wnt signaling is a potent regulator of liver gluconeogenesis [49,50]. Consistent with this observation, we found that PEDF KO mice have profoundly elevated serum glucose that is driven by elevated gluconeogenesis [48]. Restoration of PEDF dampened systemic glucose levels and suppressed key enzymes involved in gluconeogenesis. Finally, deletion of PEDF in models of pre-cancerous biology is permissive for malignancies associated with enhanced Wnt signaling including hepatocellular carcinoma and pancreatic cancer [23,51]. Thus, various functional outcomes seen in PEDF null mice *in vivo* are consistent with activation of Wnt signaling. We next turn our attention to the role of PEDF in stem cell biology and address potential therapeutic roles for PEDF involving regenerative potential.

## 4. Stem Cells

Stem cells are undifferentiated cells that are capable of self-renewal and differentiation into mature, specialized cell types [52]. Broadly, stem cells are classified into two categories based on their origins: embryonic stem cells (ESCs) and adult stem cells. Embryonic stem cells were first derived from the inner cell mass of the murine blastocyst [53], and then later produced using human embryos [54]. They are termed pluripotent, as they have the potential to differentiate into all three embryonic germ layers: ectoderm, endoderm, and mesoderm [52,55]. Maintenance of stemness and continuous growth of ESCs is enhanced by secreted factors derived from fibroblast feeder cells including PEDF [56,57].

**Figure 2:**
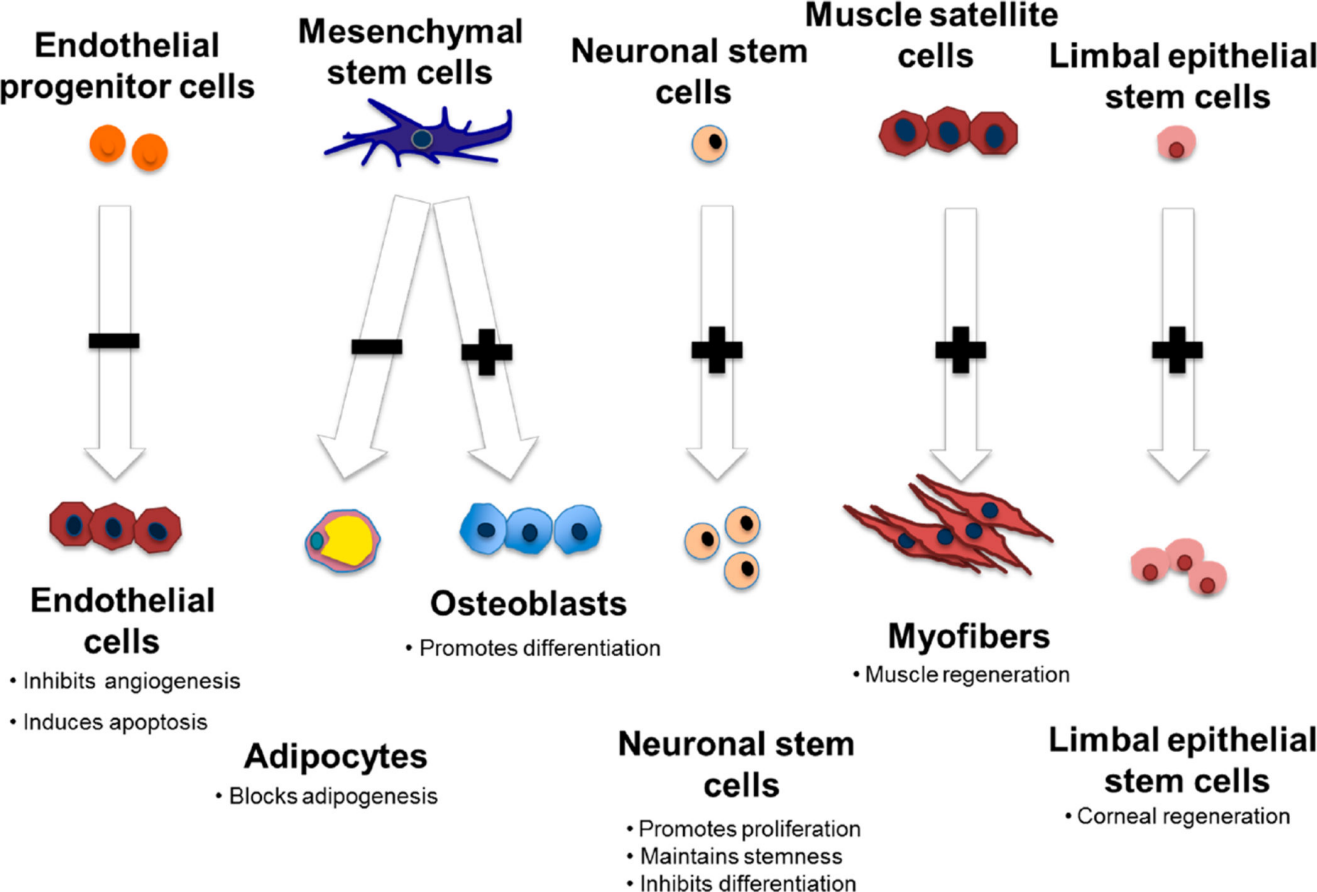
Pigment epithelium-derived factor (PEDF) regulates the proliferation and differentiation of multiple tissue-specific stem cells. PEDF targets proliferating but not resting endothelial cells. PEDF directs mesenchymal stem cells to the osteoblast lineage, while inhibiting adipogenesis. PEDF promotes neurogenesis in neuronal stem cells but causes neuronal differentiation of more committed precursors. PEDF also causes proliferation of muscle progenitors and limbal epithelial cells that are important for corneal regeneration.

PEDF has been implicated as necessary for maintenance of ESCs in the undifferentiated state and for proliferation/differentiation of multiple types of tissue resident stem cells (Figure 2). In human ESCs, PEDF was identified through an unbiased phenotypic and reporter screen to search for novel secreted proteins involved in maintaining ESC pluripotency [57]. Human H1 and H9 ESC cell lines incubated with PEDF maintained pluripotency for up to 17 generations and formed teratomas after inoculation into SCID mice [58]. Other investigators also noted that PEDF secreted by fibroblast feeder cells sustains ESCs, providing evidence to support the role of novel secreted proteins in ESC survival [56,59]. Activation of the ERK1/2 signaling pathway by PEDF is considered necessary to support hESC self-renewal and pluripotency [57]. In contrast, other reports notably find that differentiated cells derived from iPSCs can induce apoptosis of undifferentiated iPSCs through a PEDF-dependent mechanism [60].These strikingly opposing effects in two distinct stem cell populations, one supportive and the other pro-apoptotic, warrants additional investigation since there are clear implications given the entry of iPSC therapies into clinical use. The future interrogation of developmental pathways known to be affected by PEDF including Wnt and Notch pathways may be helpful to understand these differential effects.

In contrast to ESCs; adult stem cells are partially differentiated cells that are capable of differentiating into several cellular types of the tissue from which they originate. Studies have provided evidence that adult stem cells are indeed multipotent and not simply progenitors of different types of cells of their original tissue [61,62]. In the next section; we review the effects of PEDF on tissue-specific adult stem cells as well as those therapies generated from inducible pluripotent stem cells.

### 4.1. Musculoskeletal System

Mesenchymal stem cells (MSCs) are a heterogeneous collection of multipotent progenitor cells, capable of differentiating into mesodermal cells such as osteoblasts, adipocytes, chondrocytes, or myocytes. Multiple proteins, hormones, and growth factors influence the lineage specification of MSCs to different cellular lineages from MSCs [63]. Prior to the discovery of the PEDF null state in humans, it was known that PEDF was one of the most abundant proteins in the secretome of MSCs, but its role was unclear [20,64]. A high-resolution proteomic analysis of mouse MSC conditioned medium identified PEDF along with structural proteins such as collagen type 1 and fibronectin. Functionally, depletion of PEDF from the MSC secretome completely nullified fibroblast migration [64]. This striking result indicates that MSC-derived PEDF attracts fibroblasts to generate a localized niche appropriate for MSC differentiation. Thus, secretion of high levels of PEDF by MSCs and its avid binding to components of the extracellular matrix such as collagen I suggest an important role as an MSC differentiation factor.

MSCs are responsible for bone development and depend on signaling pathways such as Wnt signaling to induce osteoblast differentiation [65]. Activation of these pathways leads to concomitant blockade of competing pathways, which specify alternative lineage specification. Wnt activation of MSCs, for instance, occurs at the expense of PPARγ activation, thereby favoring osteoblast over adipocyte lineage specification [66,67]. Adding PEDF protein to MSC populations led to osteoblast lineage specification through Wnt signaling activation and marked suppression of PPARγ expression [19,21]. In its absence, MSCs fail to mineralize adequately, a reflection of impaired osteoblast differentiation. These studies therefore provide a cellular explanation as to why absence of PEDF results in a disease of impaired bone maturation characterized by inadequate mineralization. In addition, our studies also demonstrate that PEDF, like other secreted Wnt antagonists [68,69], can function as either a Wnt agonist or antagonist depending upon the cellular differentiation status of the target cell [19].

Confirmatory studies from other groups demonstrated that PEDF could direct MSCs to the osteoblast lineage and regulate other genes involved in osteogenesis [20,21]. Niyibizi and colleagues reported that supplementing exogenous PEDF to human osteoblasts led to a 47% increase in mineralization and increased expression of osteoblast-related genes compared to cells without PEDF [20]. PEDF knockdown in MSCs led to a significant decrease in osteoblast differentiation and mineralization; osteoblast differentiation was rescued by exogenous PEDF [20]. Increased expression of PEDF within proliferative and hypertrophic zones of the growth plate indicates localization in regions of active bone remodeling and diminished PEDF expression toward the base of the growth plate and in mature bone [6,70]. Thus, multiple studies now demonstrate a PEDF-directed effect on MSC to osteoblast differentiation that provides a cellular basis for explaining how the absence of PEDF results in inadequate bone formation.

The mechanisms by which PEDF promote mineralization of the bone matrix are becoming better understood. One mechanism appears to be that PEDF suppresses factors that inhibit mineralization such as sclerostin (inhibitor of Wnt signaling) and matrix constituents such as extracellular phosphoglycoprotein (MEPE). PEDF also enhanced β-catenin levels, the nuclear transcription factor for terminal Wnt signaling activation [19,21]. In our experience with PEDF null stem cells, we also find an imbalance in the extracellular matrix (unpublished results). PEDF therefore enhances expression of transcription factors that determine osteoblast differentiation while suppressing secreted factors that inhibit bone mineralization and extracellular matrix deposition.

Other mesoderm-derived tissue sites are also PEDF responsive. Skeletal muscle consists of multinucleated contractile muscle cells, called myofibers. The adult stem cells are satellite cells, which are mitotically quiescent and found in the basal lamina of myofibers. Satellite cells represent a pool of cells that can undergo proliferation in response to injury to form new myoblasts. Myoblasts then migrate to the site of injury, fully differentiate, and fuse to form new myofibers. Recently, PEDF and a synthetic peptide derived from PEDF (Ser93-Leu112) were shown to induce satellite cell proliferation and muscle regeneration *in vivo* [36]. In a rodent model of soleus muscle necrosis, PEDF and PEDF peptide increased the proliferation of satellite cells in injured muscles and promoted muscle regeneration. This was accompanied by activation of ERK1/2, Akt, and STAT3 signaling pathways [36]. These data support the notion that PEDF regulates progenitor cell populations that determine bone development and muscle regeneration.

### 4.2. Neurotrophic Effects

Self-renewal of neural stem cells (NSCs) involves maintenance and proliferation of undifferentiated cells in response to signals contained in the local environmental niche. An overview of the stem cell niche identifies NSCs in two main regions of the adult brain: the subventricular zone (SVZ) of the lateral ventricle and the subgranular zone of the dentate gyrus in the hippocampus [71,72]. At least three kinds of progenitor cells exist: B, C, and A cells [73]. Type B cells are astrocyte-like NSCs characterized by prolonged division cell cycles, and their progeny are transient-amplifying cells, neural progenitors, or C cells. C cells divide rapidly and give rise to neuroblasts, also called A-cells, which can migrate and then terminally differentiate into interneurons [74,75]. The original studies of PEDF biology noted its ability to induce neuronal differentiation of retinoblastoma cells in culture, suggesting potential effects on neuronal progenitor cells [2,27,76]. We devote the next section to PEDF’s effects on NSCs and the niche that maintains their stem-like characteristics.

Experimental evidence demonstrates that PEDF supports the renewal of stem cells in the brain. PEDF injection into the ventricles stimulated Type B cell proliferation, which did not occur with injection of the PEDF C-terminus (aa195–400). Thus, stem cell properties reside in the N terminus of the PEDF protein. In contrast, PEDF depletion reduced NSC self-renewal [29]. *In vitro*, PEDF or drugs that increase PEDF cause neurospheres to proliferate [29,77]. These effects were also relevant to murine models of accelerated senescence (SAM-P8) where NSCs demonstrated diminished self-renewal and increased neuronal differentiation properties in response to PEDF [78]. Interestingly, senescence itself has been shown to diminish levels of endogenous PEDF, although endogenous levels of PEDF were not established in SAM-P8 mice [79]. Thus, PEDF can induce neurogenesis *in vivo* whereas senescent NSCs appear unresponsive to PEDF.

These functional effects on neurogenesis by PEDF occur in conjunction with activation of developmental programs. For instance, Notch signaling promotes the self-renewal capacity of NSCs and maintains NSCs in an undifferentiated state [80]. PEDF enhances transcription factors associated with Notch signaling and the progenitor state including Hes1/Hes5 and Sox2 [29]. The mechanisms for Notch activation by PEDF involve displacement of the transcriptional co-repressor N-CoR from specific Notch-responsive promoters, and may represent a common mechanism for PEDF’s effects on stem cell populations [81,82]. Although Wnt signaling has not been specifically studied in models of PEDF-directed neurogenesis, the significant cross talk and complementary roles played by Notch and Wnt signaling in stem cell biology suggest a likely role for this pathway as well [83,84].

Clinically used drugs in neurodegenerative disorders modulate PEDF levels. For instance, PEDF increased stem cell populations in the hippocampus after injection of memantine, an NMDA receptor antagonist used in Alzheimer’s disease. Memantine injection led to strong PEDF expression in the adult hippocampus [77]. *In vitro*, PEDF significantly increased the number of large hippocampal neurospheres and impeded the differentiation of neurospheres, thereby maintaining multipotency. Neurosphere proliferation, however, occurred only in the presence of epidermal growth factor or fibroblast growth factor (FGF)-2, indicating that PEDF promotes an environment that is permissive for progenitor cell proliferation but does not induce stem cell proliferation alone [77]. These data suggest a role for PEDF and pharmacologic agents that increase PEDF in maintaining a NSC niche favorable for continued stem cell renewal.

PEDF’s role in neurogenesis and neuronal differentiation may have therapeutic implications. Secretion of PEDF by somatic stem cells derived from the umbilical cord promotes neuronal maturation, pointing to the context specific effects of PEDF [85]. In the most common brain tumor of adults, glioblastoma multiforme (GBM), a subset of cells termed glioma stem cells (GSCs) possess a gene expression profile and phenotypic characteristics resembling adult NSCs [86–90]. GSCs are promoted by factors such as epidermal growth factor receptor variant III (EGFRvIII), and this factor in turn induces the expression and secretion of PEDF [91]. PEDF sustains GSC self-renewal in a manner analogous to its effects on non-malignant NSCs through Notch-Sox2 activation [29,91]. Somewhat paradoxically, PEDF expression is inversely correlated with a histological grade of glioma and patient survival [92]. Experimental studies also indicate that PEDF diminishes glioma growth *in vivo* through anti-angiogenic and pro-apoptotic effects [58,93]. Although these inhibitory effects are consistent with PEDF’s well-known anti-tumor properties, the GSC promoting effect of PEDF suggests that tumor stem cells will be sustained by PEDF.

### 4.3. Ocular Development

Age-related macular regeneration (AMD) is a leading cause of blindness in the elderly population. AMD is characterized by degeneration and loss of retinal pigmented epithelial (RPE) cells and photoreceptor cells in the macular region [94]. Recent advances in technology to induce stem cells provide a new approach for cell replacement in this disease by applying patient-specific regenerative medicine (Schwartz SD embryonic stem cell trials 2012). Arnhold *et al.* transduced PEDF expressing MSCs through adenoviral vectors *in vitro* before sub-retinal transplantation into rats and showed that MSCs adopt RPE-like characteristics and promote the rescue of photoreceptor cells. Their findings indicated a role for PEDF in maintaining the RPE layer [95]. Other groups have similarly identified a role for PEDF in promoting the survival of retinal progenitor cells (RPCs). RPE cells derived from the H3 cell line demonstrated marked increase in PEDF secretion that enhanced the survival of RPCs [96]. Removal of secreted PEDF abrogated the survival effects derived from RPE cells [96]. PEDF, therefore, plays a critical role in the survival of cells that are retinal progenitors.

These studies have clinical relevance. Clinical trials using human MSCs (ID: NCT01531348; https://www.clinicaltrials.gov) and human neural stem cells (ID: NCT01632527; https://www.clinicaltrials.gov) to protect photoreceptors in Retinitis Pigmentosa and dry AMD, respectively, are ongoing but newer studies using induced pluripotent stem cells (iPSCs) may be more efficacious. Here, iPSCs differentiated to RPE cells have been injected into models of macular dystrophy and retinal degeneration to assess effects on cellular function and photoreceptor integrity [97,98]. RPE cells derived from iPSCs survived longer than MSCs or NSCs, possibly due to lower apoptosis rates, and diminished inflammatory cell accumulation in RPE grafts. PEDF expression in the intraocular fluid was found to be critical for inhibiting the degeneration of photoreceptors in multiple studies [97,98]. Whether PEDF has differential effects on survival of relatively differentiated retinal progenitors, while causing apoptotic cell death of undifferentiated cells thereby suppressing potential tumor or teratoma formation has been investigated [60]. In summary, PEDF secreted by RPE appears to play an important role in the regenerative potential of iPSC technologies used for diseases that lead to blindness.

Another resident stem cell population in the eye that has been the focus of PEDF biology are limbal epithelial stem cells (LSCs). LSCs are located between cornea and conjunctiva, and their asymmetric division generates transient amplifying cells that renew the corneal epithelium [99]. Cultured LSCs supplemented with PEDF or 44-mer PEDF enhanced LSC proliferation and maintained expression of limbal stem cell markers *in vitro* [100]. In a corneal injury model, 44-mer PEDF significantly promoted corneal re-epithelialization and enhanced LSC proliferation through induction of p38 MAPK and STAT3 signaling [100]. Another study found that topical 44-mer PEDF complemented these corneal repair effects by inhibiting vascularization of the corneal surface, thereby demonstrating a multi-modal capacity for PEDF in the corneal regenerative responses after injury [35].

## 5. Future Directions

Identification of PEDF null mutations as the cause of OI Type VI has defined its importance as a regulator of bone development. A number of common clinical disorders are characterized by an osteodystrophy and alterations in PEDF levels. Whether PEDF regulates bone homeostasis in these diseases warrants further investigation. Further, the PEDF null state in humans has permitted a re-examination of PEDF’s previously described effects on stem cell biology through the framework of developmental pathways.
